# Modelling the Economic Impact of lnfluenza Vaccine Programs with the Cell-Based Quadrivalent Influenza Vaccine and Adjuvanted Trivalent Influenza Vaccine in Canada

**DOI:** 10.3390/vaccines10081257

**Published:** 2022-08-04

**Authors:** Van Hung Nguyen, Bertrand Roy

**Affiliations:** 1 VHN Consulting Inc., 95 McCulloch, Montreal, QC H2V3L8, Canada; 2 Seqirus Canada, 16766 Trans-Canada Hwy Suite 504, Kirkland, QC H9H 4M7, Canada

**Keywords:** influenza, cell-based influenza vaccine, Canada, cost-effectiveness, ICER

## Abstract

In Canada, approximately 12,000 people annually are hospitalized with influenza. While vaccination is the most effective method for reducing the burden of seasonal influenza, the propagation of vaccine virus strains in eggs can result in egg adaption, resulting in reduced antigenic similarity to circulating strains and thus lower vaccine effectiveness (VE). Cell-based propagation methods avoid these alterations and therefore may be more effective than egg-propagation vaccines. We evaluated three different scenarios: (1) egg-based quadrivalent influenza vaccine (QIVe) for individuals <65 years and adjuvanted trivalent influenza vaccine (aTIV) for ≥65 years; (2) QIVe (<65 years) and high-dose QIV (HD −; QIV; ≥65 years); and (3) cell-based derived QIV (QIVc; <65 years) and aTIV (≥65 years) compared with a baseline scenario of QIVe for all age groups. Modelling was performed using a dynamic age-structured SEIR model, which assessed each strain individually using data from the 2012–2019 seasons. Probabilistic sensitivity analysis assessed the robustness of the results with respect to variation in absolute VE, relative VE, number of egg-adapted seasons, and economic parameters. QIVe + aTIV was cost-saving compared with the baseline scenario (QIVe for all), and QIVe + HD − QIV was not cost-effective in the majority of simulations, reflecting the high acquisition cost of HD − QIV. Overall, while the incremental benefits may vary by influenza season, QIVc + aTIV resulted in the greatest reductions in cases, hospitalizations, and mortality, and was cost-effective (ICER < CAD 50,000) in all simulations.

## 1. Introduction

Influenza is one of the leading causes of morbidity and mortality in Canada, associated with approximately 12,200 hospitalizations and 3500 deaths annually [1,2,3]. Annual vaccination is the most effective method for prevention of seasonal influenza, benefiting both vaccinated individuals and reducing transmission to other vulnerable community members [4,5]. In Canada, vaccination is highly recommended for all children aged 6–59 months, adults and children with underlying chronic health conditions, pregnant women, nursing home residents, adults >65 years of age, and individuals who are at increased risk of transmitting the disease to vulnerable populations [6]. However, 9 out of 10 provinces have universal influenza immunization programs that recommend vaccination to all individuals 6 months and older with the age-appropriate influenza vaccine [7]. Currently available types of seasonal influenza vaccine include standard-dose trivalent (TIV) and quadrivalent (QIV) formulations, providing protection against A/H1N1, A/H3N2, and one (TIVs) or both (QIVs) B strains; adjuvant TIVs or high-dose QIVs; and a live attenuated quadrivalent influenza vaccine nasal spray (LAIV). Recommended vaccines vary by age group, with QIVs recommended in infants aged 6–23 months, QIVs or LAIV for children aged 2–17 years, and standard-dose TIVs, QIVs, or LAIV for adults <65 years [6]. As vaccine effectiveness (VE) is lower in adults ≥65 years due to age-related immunosenescence [8], adjuvanted and high-dose formulations are also recommended for this age group, although availability of vaccine types may vary by region or province. 

In recent seasons, the effectiveness of influenza vaccines has decreased against the A/H3N2 strain, particularly in seasons where there have been antigenic differences between the circulating and vaccine strains [9,10,11]. While influenza transmission has been significantly reduced by public health measures implemented during the COVID-19 pandemic [12], preliminary analysis of the pre-COVID-19 pandemic 2019–2020 influenza season in Canada estimated VE across all age groups as 44% against A/H1N1pdm09, 69% against B, and 62% effectiveness against the A/H3N2 strain [13]. However, in a meta-analysis of 56 studies of PCR-confirmed influenza, pooled VE across ages for the overall study populations was estimated as 33% (95% confidence interval (CI): 26–39%) against A/H3N2, compared with 54% (46–61%) and 61% (57–65%) against the B and A/H1N1pdm09 strains, respectively [9]. This decreased VE against A/H3N2 has been at least partially linked to viral egg-adaptation mutations in the haemagglutinin (HA) surface glycoprotein, arising during propagation of the vaccine strain [14,15]. 

While most seasonal influenza vaccines available globally are produced by growing virus strains in eggs, propagation of influenza vaccine strains in mammalian cell lines, such as Madin–Darby canine kidney (MDCK) cells, avoids these egg-based mutations and provides a closer match to the WHO-mandated strains. In analyses of the 2018–2019 influenza seasons in the United States, individuals aged 4 years and over vaccinated with a cell-based QIV (QIVc) had a greater reduction in influenza-related medical encounters than those vaccinated with an egg-based QIV (QIVe; relative VE (rVE): 7.6%) [16], with a separate study of the 2017–2018 season showing an adjusted rVE of 36.2% for prevention of influenza-related medical encounters [17]. A QIVc (Flucelvax^®^ Quadrivalent, Seqirus Inc, Holly Springs, CA, USA) was first introduced in Canada in 2019 for persons aged 9 years and older and has demonstrated non-inferiority to cell-based TIVs in paediatric and adult clinical trials, with a favourable safety profile [18,19]. It recently received an expanded age indication of 6 months and older.

Given the potential for higher VE against A/H3N2 by avoiding egg-adaptation mutations, we evaluated the cost-effectiveness of different QIVc vaccination scenarios in combination with enhanced vaccines (adjuvanted or high-dose) for individuals ≥65 years compared with QIVe in the Canadian population. 

## 2. Methods

### 2.1. Model Structure and Parameters

The epidemiological model used in this analysis was an age-structured four-strain dynamic transmission model previously used to model influenza epidemiology in the US [20]. In brief, estimates of influenza incidence were combined with virological data to estimate yearly incidence per strain for Canada, with assumptions of 66% of infected individuals being symptomatic, an incubation period of 0.8 days, and an infectious period of 1.8 days. 

**Figure 1:**
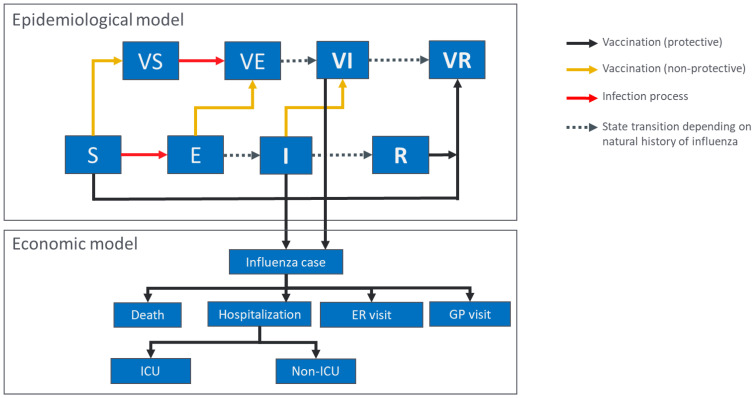
Outline of the epidemiological and economic model structures. S, E, I, and R represent susceptible, exposed, infectious, and recovered individuals, respectively, for the individual influenza strains. VS, VE, and VI represent susceptible, exposed and infectious individuals who received an influenza vaccine which was non-protective for the strain in question; VR represents individuals who were vaccinated and protected against influenza (either through infection following non-protective vaccination or through vaccination). In the economic model, infected individuals are categorized based on their healthcare usage. ER, emergency room; GP, general practitioner; ICU, intensive care unit.

The model used was a classic SEIR model, as often used for modelling influenza transmission for evaluating vaccination programs (e.g., [21,22]), where the population was assumed to be either susceptible to infection (S), exposed to the virus (E), infected and infectious (I), or recovered from infection (R) (Figure 1). Vaccination was modelled by removing a select number of individuals (VR) from the susceptible compartment immediately following administration of the vaccine. We assumed that this group was fully protected against influenza infection, whereas the remaining fraction of vaccinated individuals received no protection (VS) and hence still contributed to the infection dynamic. The model was used to generate simulations of seasonal influenza epidemics based on historical data from 2012–2019 [23]. Mixing of the population was based on POLYMOD data for Canada [24]. Epidemiological dynamics were independently simulated for each of the influenza strains, i.e., A/H1N1pdm09, A/H3N2, B/Victoria, and B/Yamagata. The model was structured by age group based on 16 age categories (6–23 months, 2–3 years, 4–8 years, 9–17 years, 18–24 years, 25–29 years, 30–35 years, 36–39 years, 40–44 years, 45–49 years, 50–54 years, 55–59 years, 60–64 years, 65–69 years, 70–74 years, and ≥75 years; Supplementary Table S1). Birth and death rates were set as equal to maintain a constant population size and population distribution among age classes.

Data on circulating influenza strains from 2012 to 2019 were obtained from Flunet, using specific data for Canada [23]. As no specific incidence rate data were available for Canada, these values were used to generate calibrated attack rates specific to strains based on disease-burden data from the US from 2012 to 2019 (Supplementary Table S2) [25]. Each year was categorized as being a matched or unmatched year for each individual vaccine strain [26] (see Table 1). VE measures per strain for those years were obtained from the SPSN network [27]. Relative VE (rVE) of QIVc vs. QIVe was estimated using data pooled from retrospective studies for the 2017–2019 influenza seasons [16,17,28,29,30]. The rVE per strain was calculated assuming only that the A/H3N2 strain was unmatched during egg-adapted years in using the method described in the US study [20]. rVE was assumed to be constant across age groups, with an extra assumption that available data for estimating rVE (based on individuals ≥4 years of age) was also applicable to children <4 years of age. 

The economic model was based on that described in Fisman et al. [31], using the same input parameters as presented in Table 2. Similar to the Fisman model, the age-specific impact of influenza was assessed as estimated probability of healthcare utilization visits multiplied by the number of resource units and the unit-cost of each visit. The number of cases estimated from the epidemiological model were used as an input for the economic model to estimate the other outcomes, such as hospitalization and death. Vaccine price parameters were estimated using the product list price [32], with an assumption that 50% of children <3 years of age would require two doses. Although Canada does not have a formal incremental cost-effectiveness ratio (ICER) threshold, we assumed that an ICER < CAD 50,000 per quality-adjusted life year (QALY) would be cost-effective. The time horizon used was 2012–2019, during which there were six egg-adapted seasons (2012–2014, 2016, 2017, and 2019). Six egg-adapted seasons were used as the baseline scenario, with sensitivity analysis (see below) performed based on only three egg-adapted seasons which were randomly selected as part of the model from the eight possible seasons on the time horizon. As the model used historical epidemiological data for the years 2012 to 2019, for predictions of the future, a discounted rate of 5% was applied to a time horizon of 8 years. 

### 2.2. Scenarios

Three scenarios were compared to a standard baseline scenario of QIVe for all age groups, assuming six unmatched influenza seasons and an rVE for A/H3N2 of 15.6%, as estimated from the pooled retrospective studies analysis for the 2017–2018 seasons. Scenario 1 evaluated the use of QIVe for all individuals aged 6 months to 64 years and adjuvanted TIV (aTIV) for adults ≥65 years. Scenario 2 replaced aTIV with high-dose QIVe (HD-QIV) for adults ≥65 years. Scenario 3 evaluated QIVc for 6-month-olds to 64-year-olds and aTIV for adults ≥65 years. Individuals <65 years of age were assumed to receive the same vaccine, irrespective of whether they were considered low or high risk for influenza. Coverage rates by age group are shown in Supplementary Table S3 [33]. Additional analysis was performed using different A/H3N2 rVE scenarios of 7.6% (derived from pooled data of retrospective cohort studies from 2018–2019 [16,29]) and a mix of both values to reflect the heterogeneity of vaccine efficacy by season. In the mixed scenario, 15.6% was used for seasons with high levels of circulation of A/H3N2 (2012, 2014, 2016, 2017) and 7.6% for seasons with low circulation of A/H3N2 (2013 and 2019). For the 2015 and 2018 seasons, which were not reported as egg-adapted years, the rVE was set as 0. 

### 2.3. Sensitivity Analysis

Stochastic probability sensitivity analysis was performed on vaccine effectiveness, season profile (i.e., which seasons were matched/unmatched), economic inputs, and the number of egg-adapted seasons. For the simulation, 500 sets of parameters were randomly drawn from a normal distribution, as described previously by Fisman et al. [31]. A ±10% variation around the estimated assumption in costs was evaluated, with VE and rVE drawn from their corresponding 95% confidence intervals (CIs) and assessments performed for six or three egg-adapted influenza seasons. The results are presented as averages across influenza seasons. 

## 3. Results

### 3.1. Epidemiological Impact

**Figure 2:**
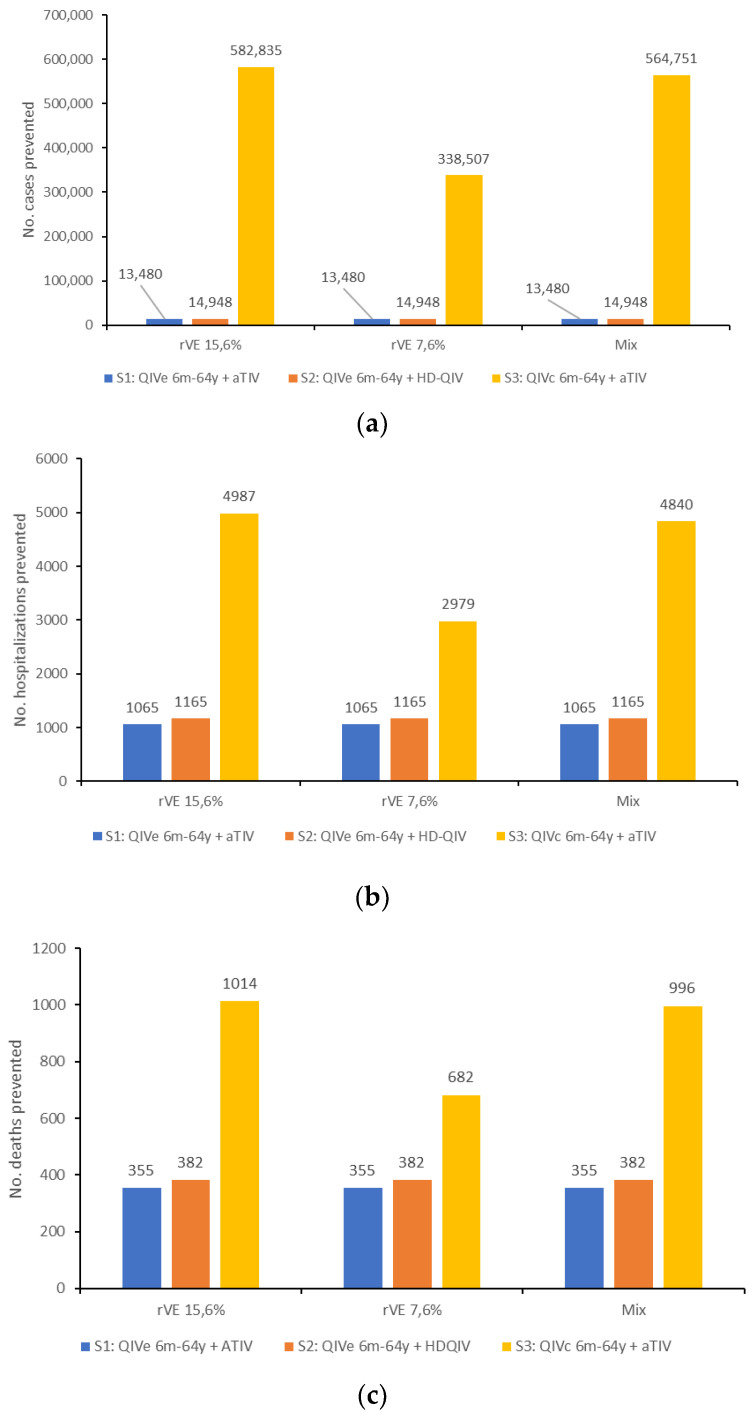
(**a**) Number of cases, (**b**) number of hospitalizations, and (**c**) number of deaths prevented by each vaccine scenario, compared with the baseline scenario, for each of the rVE values evaluated.

All three scenarios assessed against the baseline scenario resulted in reduced cases, hospitalizations, and deaths from influenza (Table 3). The greatest reductions were seen for scenario 3, i.e., QIVc for recipients <65 years of age and aTIV for recipients ≥65 years of age, preventing up to 582,835 cases, 4987 hospitalizations, and 1014 deaths (Figure 2). In scenarios 1 (QIVe for <65 years and aTIV for ≥65 years) and 2 (QIVe for <65 years and HD-QIV for ≥65 years), much smaller impacts were seen on the numbers of cases prevented (13,480–14,948), although the impacts on hospitalizations and deaths were still substantial (1065–1165 and 355–382, respectively), mainly due to the fact that individuals ≥65 years have a greater probability of hospitalization or death than younger age groups.

### 3.2. Economic Impact

Scenario 1 (QIVe+aTIV) was cost-saving compared with the base case scenario, with total direct savings of approximately CAD 20 million (Table 3). Scenarios 2 (QIVe+HD-QIV) and 3 (QIVc+aTIV) resulted in reduced medical costs of approximately CAD 24 million and CAD 103 million, respectively, but the higher costs of the vaccines resulted in increased direct costs of approximately CAD 278 million and CAD 20 million, respectively. 

Assessment of the ICER across different rVE estimates showed that scenario 1 was cost-saving with all three rVE estimates, whereas scenario 2 was above the CAD 50,000 threshold in all three cases (Table 4). Scenario 3 was cost-effective across all three rVE estimates, with ICERs ranging from CAD 1300 to CAD 6900. 

**Figure 3:**
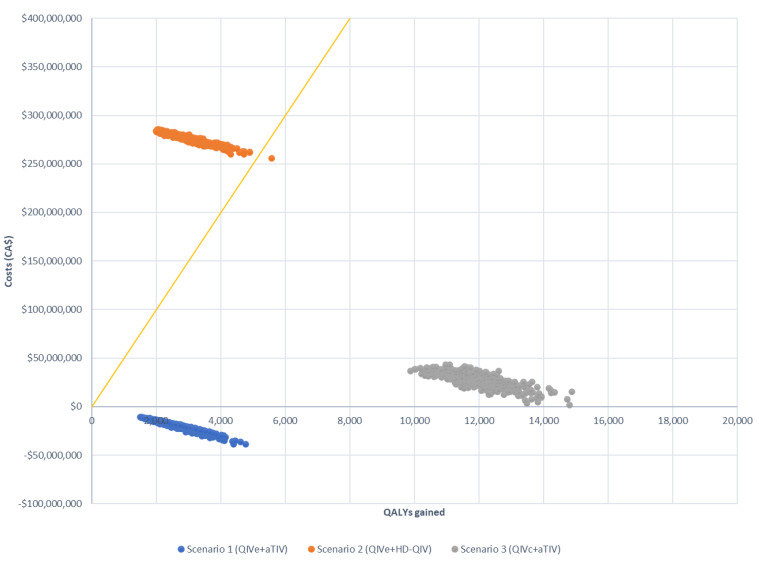
Probabilistic sensitivity analysis of three scenarios compared with the baseline (QIVe for all age groups), assuming six egg-adapted seasons. Costs are presented as CAD. The yellow line indicates the willingness to pay the threshold (CAD 50,000).

**Figure 4:**
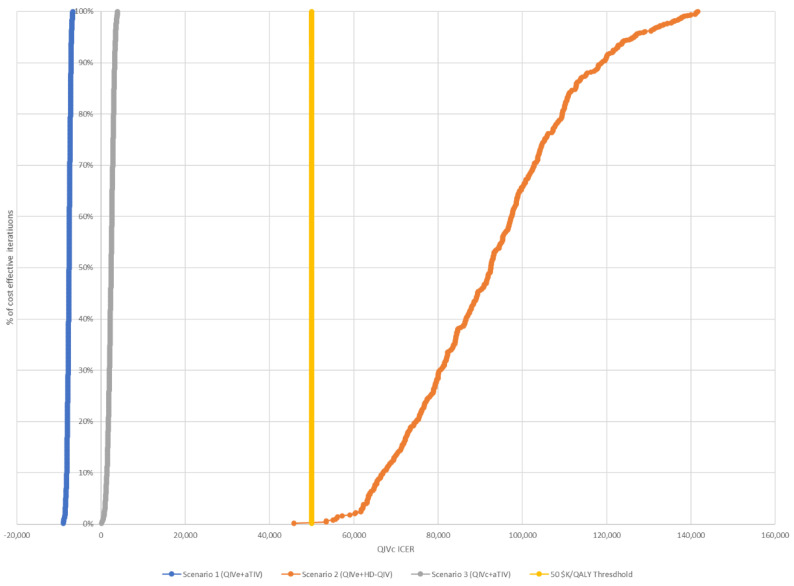
Cost-effectiveness acceptability curves for each of the three scenarios, assuming six egg-adapted seasons. The threshold for cost-effectiveness was CAD 50,000.

These findings were confirmed by sensitivity analysis, which showed that scenario 1 remained cost-saving and that the upper bound of the 95% CI remained under the cost-effectiveness threshold for scenario 3 in all situations assessed (Table 5). Scenario 2 was not cost-effective in any of the situations evaluated. Based on the assumption of six egg-adapted seasons, all simulations were cost-effective for scenarios 1 and 3; however, the majority of simulations exceeded the CAD 50,000 threshold for scenario 2 (Figure 3 and Figure 4). Similar results were observed for simulations using only three egg-adapted seasons (Supplementary Figures S1 and S2).

## 4. Discussion

While high levels of antigenic similarity have been observed worldwide for the past 15 seasons (both in the Northern Hemisphere and in the Southern Hemisphere) between vaccine and circulating A/H1N1 strains, egg adaptation of the A/H3N2 vaccine strain is of particular concern for seasonal influenza vaccination [26]. Adaption to egg propagation structurally alters the haemagglutinin receptor binding site, resulting in antigenic differences to circulating virus strains [14,15]. Vaccine-induced antibody responses generated against these egg-based strains are lower than those against the circulating strain, resulting in reduced VE and lower protection from infection and serious illness [26]. In addition, the A/H3N2 strain is responsible for the highest number of hospitalizations and deaths in individuals ≥65 years of age [34,35], a group particularly vulnerable to influenza due to immunosenescence, which results in decreased immune system function and reduced ability to develop immunity following vaccination [36,37]. As enhanced vaccines such as aTIV have shown higher effectiveness in older adults than standard-dose vaccines [38,39], the combination of an enhanced vaccine for adults ≥65 years together with a QIVc for younger age groups may provide the highest population-level protection against A/H3N2. 

Poor VE against A/H3N2, together with the challenges of limited immune response in older adults, has led many countries to routinely recommend enhanced vaccines (e.g., adjuvanted or high-dose vaccines) for seasonal use in older adults. In Canada, enhanced vaccines are among the vaccines recommended to be offered to adults aged ≥65 years, although standard-dose and unadjuvanted vaccines are also recommended [6]. In contrast, in the UK, an adjuvanted QIV (aQIV) is recommended for all adults aged ≥65 years, with QIVc recommended for individuals <65 years of age [40]. aQIV is also recommended for adults ≥65 years in Australia, with QIVe or QIVc for individuals aged 6 months (from 2 years for QIVc) to <65 years [41]. 

Egg adaption appears to have an important impact on the effectiveness of vaccines against A/H3N2. Analysis in recent years has shown multiple influenza seasons where the circulating A/H3N2 strain was antigenically different to the vaccine virus in QIVe vaccines, whereas this difference was less likely to occur with QIVc vaccines: across the 2012–2016 seasons, 20% of egg-based and 100% of cell-based A/H3N2 vaccine viruses were antigenically similar to circulating strains [26]. Overall, there was little or no antigenic similarity between A/H3N2 isolates and egg-based reference viruses, as assessed by hemagglutination assay, in 16 (55%) of the 29 seasons evaluated [26]. While antigenic drift still has a major impact on VE, egg adaptation is well-documented and is likely to reduce VE, particularly against A/H3N2. On average, it is thought that egg adaptation potentially reduces influenza VE by 4–16%, with the highest impacts on the A/H3N2 strain in individuals aged <65 years [42]. However, the degree to which egg adaptation affects VE is still to be robustly evaluated, though it is likely that the degree and frequency of occurrence of egg adaptation is underestimated, as only limited samples have been analyzed to date [26]. 

Several studies over the past three Northern Hemisphere seasons have demonstrated an increased effectiveness of QIVc compared with standard-dose egg-based vaccines, although absolute VE estimates vary by season, in line with the unpredictability of circulating A/H3N2 strains. Part of this observed variability is due to differences in study designs, with retrospective cohort studies showing significantly increased effectiveness of QIVc over QIVe, whereas only trends with large confidence intervals were observed with test negative design studies [16,17,28,43,44]. One of the limitations of test negative design studies is that they lack statistical power, which may explain the large confidence intervals and lack of statistically significant differences in VE between the vaccines in this type of study [45]. The current analysis was performed using the results from a retrospective cohort design for QIVc for this reason. Additionally, recent systematic reviews have demonstrated the increased effectiveness of HD-QIV and adjuvanted vaccines compared with standard-dose vaccines, indicating the advantage of both types of vaccine for older individuals [38,39,46]. In our analysis, we assumed the same VE for both vaccines and therefore did not directly assess the differences between identical scenarios containing HD-QIV versus aTIV. This assumption is corroborated by the recent ACIP systematic review of data available for the 65+ population, where it was concluded that there is no difference in effectiveness between the enhanced vaccines, assuming there is no mismatch in B strains [47]. Only when there is a mismatch with B strains will HD-QIV provide a higher protection against B strains, since it contains both B strains. However, the higher cost of an individual dose of a HD-QIV vaccine underpinned the cost-effectiveness analysis, with none of the QIVe+HD-QIV situations being cost-effective, despite improvements in terms of case numbers, hospitalizations, and deaths compared with the baseline scenario.

While administration of QIVe to all ages may have a limited budget impact, the economic model confirmed that a combination of QIVc and aTIV would be cost-effective in all of the simulations assessed due to lower acquisition vaccine costs and the incremental efficacy in preventing medical visits, hospitalizations, and deaths in the most at-risk populations. This estimate is robust, as we used data collected over several seasons, which allowed us to capture the heterogeneity of influenza epidemiology from one season to another. This approach increased the external validity of the results, as it captured the main uncertainty of the variables that impact the results, i.e., the number of egg-adapted seasons and varying VE. However, there were a number of limitations to our analysis. Although we considered VE by strain, we did not vary VE by age group, and there are currently no data available on QIVc in infants and children <4 years of age. Additionally, the data used are based on the US influenza seasons from 2012–2019 and therefore may not be broadly generalizable across countries with different influenza dynamics or population structures. Thirdly, we did not evaluate the impact of aQIV in older adults, as it is not currently available in Canada. The use of aQIV compared with aTIV would potentially provide increased protection against influenza in this high-risk population and therefore may increase cost-effectiveness estimates when it becomes available in future seasons. Finally, our base-case scenario assumed six egg-adapted seasons out of the eight analyzed. While sensitivity analysis still indicated the cost-effectiveness of the QIVc+aTIV scenario with three egg-adapted seasons and with varying VE, these results may have been different with fewer egg-adapted seasons. In the unlikely scenario of only one egg-adapted season in the eight seasons evaluated, QIVc+aTIV would no longer have been cost-effective. 

In summary, this analysis has shown that vaccination of 6-month- to 64-year-olds with a cell-based QIV together with aTIV for ≥65-year-olds is cost-effective across varying assumptions of rVE and numbers of egg-adapted influenza seasons. Overall, this vaccine combination resulted in the greatest reductions in cases, hospitalizations, and deaths due to influenza compared with the other scenarios evaluated. While the incremental advantages of QIVc+aTIV will vary between individual influenza seasons, sensitivity analysis reveals that this vaccine combination would be favourable in nearly all scenarios.

## Figures and Tables

**Table 1:** Parameters used in the epidemiological model.

Year	Matching Assumptions				Absolute QIVe Vaccine Effectiveness per Influenza Strain and per Year				rVE QIVc When Egg- Adapted *	rVE HD-QIV-aTIV When Egg-Adapted *	rVE HD-QIV-aTIV When Matched
	A/ H1N1	A/ H3N2	BVIC	BYAM	A/H1N1	A/H3N2	BVIC	BYAM	A/ H3N2	A/H3N2	A and B
2012	M	U	M	U	59% (53–65%)	41% (37–45%)	68% (61–75%)	68% (61–75%)	15.6% (7–20%)	9% (7.20–10%)	24% (9.70–36%)
2013	M	U	U	M	71% (64–78%)	66% (59–73%)	72% (65–79%)	72% (65–79%)			
2014	M	U	U	M	9% (8–10%)	9% (8–10%)	9% (8–10%)	9% (8–10%)			
2015	M	M	U	M	43% (39–47%)	44% (40–48%)	50% (45–55%	50% (45–55%			
2016	M	U	M	U	36% (32–40%)	36% (32–40%)	72% (65–79%)	72% (65–79%)			
2017	M	U	M	U	58% (52–64%)	14% (13–15%	46% (41–51%)	46% (41–51%)			
2018	M	M	M	U	67% (60–74%)	17% (15–19%)	72% (65–79%)	72% (65–79%)			
2019	M	U	M	U	43% (39–47%)	50% (45–55%	65% (59–72%)	65% (59–72%)			

BVIC, B strain, Victoria lineage; BYAM, B strain, Yamagata lineage; M, matched; QIVc, cell-based quadrivalent influenza vaccine; QIVe, egg-based quadrivalent influenza vaccine; rVE, relative vaccine effectiveness; U, unmatched. * rVE was calculated and adjusted for the A/H3N2 strain during egg-adapted years using the method described and adapted from [20]. rVE estimates were calculated per strain across age groups.

**Table 2:** Parameters used in the economic model adapted from [31].

Age Group	Hospitalization ^a^	ICU ^b^	Mechanical Ventilation ^c^	ECMO ^c^	Death ^b^	GP Visit Costs ^d^ (CAD)	ED Costs ^e^ (CAD)	Hospitalization Costs (CAD)	ICU Cost (CAD)	ICU and Mechanical Ventilation Cost (CAD)	ICU and ECMO (CAD)	QALY per Case	Death Discounted (5%)
0–4 Y	0.089%	12%	81%	4.00%	1%	52.61	313.43	5103	33,242	50,411	151,726	0.985	18.53
5–19 Y	0.018%	11%	81%	4.00%	2%	44.86	286.79	6075	28,654	50,552	235,899	0.985	18.15
20–64 Y	0.033%	23%	81%	4.00%	10%	44.14	314.42	9557	20,239	61,290	96,211	0.98	15.14
65+	0.132%	16%	81%	4.00%	19%	56.29	389.74	11,894	22,164	57,084	95,684	0.97	2.41

^a^ Conditional to symptomatic case. ^b^ Conditional to hospitalization. ^c^ Conditional to ICU admission. ^d^ Assumption that 10% of cases resulted in GP visits, across age groups. ^e^ Assumption that 2.5% of cases resulted in ER visits, across age groups. Costs are given in Canadian dollars. Age groups for the economic model were based on those used in the Fisman et al. study and applied over the 16 age groups used in the current model. ECMO, extracorporeal membrane oxygenation; ED, emergency department; GP, general practitioner; ICU, intensive care unit; QALY, quality-adjusted life years; Y, years.

**Table 3:** Base case results and differences to the reference scenario (QIVe for all age groups).

	Absolute Value				Difference vs. Reference Scenario		
	Reference Scenario (QIVe for All)	QIVe (6 m to 64 y) + aTIV for ≥65 y	QIVe (6 m to 64 y) + HD-QIV for ≥65 y	QIVc (6 m to 64 y) + aTIV for ≥65 y	QIVe (6 m to 64 y) + aTIV for ≥65 y	QIVe (6 m to 64 y) + HD-QIV for ≥65 y	QIVc (6 m to 64 y) + aTIV for ≥65 y
Symptomatic influenza cases	2,793,715	2,691,577	2,681,540	2,210,880	−102,138	−112,175	−582,835
GP consultations	382,372	368,892	367,424	308,371	−13,480	−14,948	−74,001
ED consultations	95,593	92,223	91,856	77,093	−3370	−3737	−18,500
Total number of hospitalizations	22,835	21,770	21,670	17,848	−1065	−1165	−4987
Total number of ICU hospitalizations	4575	4376	4357	3585	−199	−218	−990
Total number of deaths	3379	3024	2997	2365	−355	−382	−1014
Cost of influenza vaccine (CAD)	201,324,565	203,130,422	501,893,579	325,232,342	1,805,857	300,569,014	123,907,777
Cost of influenza vaccine (discounted) (CAD)	170,782,959	172,314,862	425,754,654	275,893,514	1,531,903	254,971,695	105,110,555
Cost of medical consultations (CAD)	17,381,968	16,735,367	16,666,491	13,972,659	−646,601	−715,477	−3,409,309
Cost of hospitalizations (CAD)	458,359,891	437,223,795	435,228,411	358,292,003	−21,136,096	−23,131,480	−100,067,888
Total medical cost (CAD)	475,741,859	453,959,161	451,894,901	372,264,662	−21,782,698	−23,846,958	−103,477,197
Total medical cost (discounted) (CAD)	401,650,258	383,744,102	381,901,843	313,426,249	−17,906,156	−19,748,415	−88,224,009
Total direct cost (vaccine, medical, and hospitalizations) (CAD)	677,066,424	657,089,584	953,788,480	697,497,004	−19,976,840	276,722,056	20,430,580
Total direct cost (discounted) (CAD)	572,433,217	556,058,964	807,656,497	589,319,763	−16,374,253	235,223,280	16,886,546
QALY loss from symptomatic cases	48,901	46,849	46,657	38,340	−2052	−2244	−10,561
QALY loss from deaths	17,150	15,993	15,901	12,666	−1157	−1249	−4484
Total QALY loss	66,051	62,842	62,559	51,006	−3209	−3492	−15,045
Total QALY loss (discounted)	55,804	53,164	52,911	42,958	−2640	−2893	−12,846

aTIV, adjuvanted trivalent influenza vaccine; ED, emergency department GP, general practitioner; HD-QIV, high-dose quadrivalent influenza vaccine; ICU, intensive care unit; m, months; QALY, quality-adjusted life years; QIVc, cell-based quadrivalent influenza vaccine; QIVe, egg-based quadrivalent influenza vaccine; y, years. Costs are given in Canadian dollars.

**Table 4:** Mean ICER estimates for each scenario across the relative vaccine effectiveness estimates.

Scenario	S1: QIVe 6 m–64 y + aTIV	S2: QIVe 6 m–64 y + HD-QIV	S3: QIVc 6 m–64 y + aTIV
rVE 15.6%	Cost-saving	CAD 81,300/QALY	CAD 1300/QALY
rVE 7.6%	Cost-saving	CAD 81,300/QALY	CAD 6900/QALY
Mix ^a^	Cost-saving	CAD 81,300/QALY	CAD 1500/QALY

^a^ The mixed scenario used rVE 15.6% in seasons with high circulation of A/H3N2 (2012, 2014, 2016, and 2017) and 7.6% in seasons with low A/H3N2 circulation (2013 and 2019). ICERs are given in Canadian dollars. The time horizon for estimates included the six egg-adapted seasons (2012–2014, 2016–2017, and 2019).

**Table 5:** Probabilistic sensitivity analysis of ICER based on varying vaccine effectiveness, season profile, and number of egg-adapted seasons.

Parameter	Scenario	ICER per QALY Gained (Median)	Lower Bound 95% CI	Upper Bound 95% CI
**VE** ^a^	QIVe 6 m-64 y + aTIV ≥ 65 y	Dominant strategy	Dominant strategy	Dominant strategy
	QIVe 6 m-64 y + HD-QIV ≥ 65 y	CAD 92,994	CAD 68,503	CAD 140,674
	QIVc 6 m-64 y + aTIV ≥ 65 y	CAD 1475	CAD 431	CAD 2904
**Season Profile** ^b^	QIVe 6 m-64 y + aTIV ≥ 65 y	Dominant strategy	Dominant strategy	Dominant strategy
	QIVe 6 m-64 y + HD-QIV ≥ 65 y	CAD 89,805	CAD 81,517	CAD 98,930
	QIVc 6 m-64 y + aTIV ≥ 65 y	CAD 2479	CAD 1160	CAD 4845
**VE and Season Profile**	QIVe 6 m-64 y + aTIV ≥ 65 y	Dominant strategy	Dominant strategy	Dominant strategy
	QIVe 6 m-64 y + HD-QIV ≥ 65 y	CAD 90,670	CAD 53,355	CAD 149,047
	QIVc 6 m-64 y + aTIV ≥ 65 y	CAD 2764	CAD 891	CAD 5449
**Only Three Seasons Egg-Adapted with VE**	QIVe 6 m-64 y + aTIV ≥ 65 y	Dominant strategy	Dominant strategy	Dominant strategy
	QIVe 6 m-64 y + HD-QIV ≥ 65 y	CAD 72,879	CAD 50,288	CAD 128,852
	QIVc 6 m-64 y + aTIV ≥ 65 y	CAD 7770	CAD 3650	CAD 17,079

^a^ VE was randomly drawn from the confidence intervals presented in Table 1. ^b^ Season profile refers to matched vs. unmatched years for A/H3N2, randomly simulated across the eight seasons. aTIV, adjuvanted trivalent influenza vaccine; CI, confidence interval; HD-QIV, high-dose quadrivalent influenza vaccine; m, months; QIVc, cell-based quadrivalent influenza vaccine; QIVe, egg-based quadrivalent influenza vaccine; VE, vaccine effectiveness; y, years. ICERs are given in Canadian dollars.

## Data Availability

The CDC data analyzed in this study are available online at: https://www.cdc.gov/flu/weekly/pastreports.htm, accessed on 15 March 2022.

## Supplementary Materials

The following are available online at https://www.mdpi.com/article/10.3390/vaccines10081257/s1, Table S1: 2021 population estimates in Canada per age group; Table S2: Calibrated influenza attack rate for individual influenza strains, based on the observed strain observation in Canada and incidence rates in the US during each year; Table S3: Assumed vaccine coverage rates for the general population and high-risk individuals, by age group Figure S1: Probabilistic sensitivity analysis of three scenarios compared with baseline (QIVe for all age groups) assuming three egg-adapted seasons. Costs are presented in Canadian dolllars; Figure S2: ICER acceptability curve for each of the three scenarios, assuming three egg-adapted seasons. Threshold for cost-effectiveness was CA $50,000.
