# Supplementary material for: Modelling the Economic Impact of lnfluenza Vaccine Programs with the Cell-Based Quadrivalent Influenza Vaccine and Adjuvanted Trivalent Influenza Vaccine in Canada
Source: Vaccines (Basel). 2022 Aug 4;10(8):1257. doi: 10.3390/vaccines10081257 (PMC9412987; doi:10.3390/vaccines10081257)

Supplementary Materials

Supplementary Table S1. 2021 population estimates in Canada per age group.

Age Group	Population
6–23 months	548,840
2–3 years	761,582
4–8 years	2,022,787
9–17 years	3,717,961
18–24 years	3,298,064
25–29 years	2,640,110
30–34 years	2,697,788
35–39 years	2,666,647
40–44 years	2,509,664
45–49 years	2,384,473
50–54 years	2,430,334
55–59 years	2,700,181
60–64 years	2,606,885
65–69 years	2,233,993
70–74 years	1,853,536
75+ years	2,994,263

Supplementary Table S2. Calibrated influenza attack rate for individual influenza strains, based on the observed strain observation in Canada and incidence rates in the US during each year [23,25].

Year	A/H1N1	A/H3N2	B Victoria	B Yamagata	TOTAL
2012	0.95%	6.19%	0.85%	0.48%	8.48%
2013	5.15%	0.33%	1.03%	0.94%	7.43%
2014	0.08%	5.88%	1.41%	0.00%	7.37%
2015	3.82%	0.41%	1.11%	0.51%	5.85%
2016	0.06%	6.16%	0.58%	0.23%	7.03%
2017	0.57%	4.97%	3.86%	0.47%	9.87%
2018	4.48%	2.10%	0.27%	0.10%	6.94%
2019	3.30%	1.51%	3.48%	0.06%	8.34%

Supplementary Table S3. Assumed vaccine coverage rates for the general population and high-risk individuals, by age group.

Age Group	General Population	High Risk
6–23 months	29%	29%
2–3 years	29%	29%
4–8 years	29%	29%
9–17 years	29%	29%
18–24 years	29%	29%
25–29 years	29%	29%
30–34 years	29%	29%
35–39 years	29%	29%
40–44 years	29%	29%
45–49 years	29%	29%
50–54 years	29%	29%
55–59 years	47%	47%
60–64 years	47%	47%
65–69 years	75%	75%
70–74 years	75%	75%
75+ years	75%	75%

Supplementary Figure S1. Probabilistic sensitivity analysis of three scenarios compared with baseline (QIVe for all age groups) assuming three egg-adapted seasons. Costs are presented in Canadian dolllars.

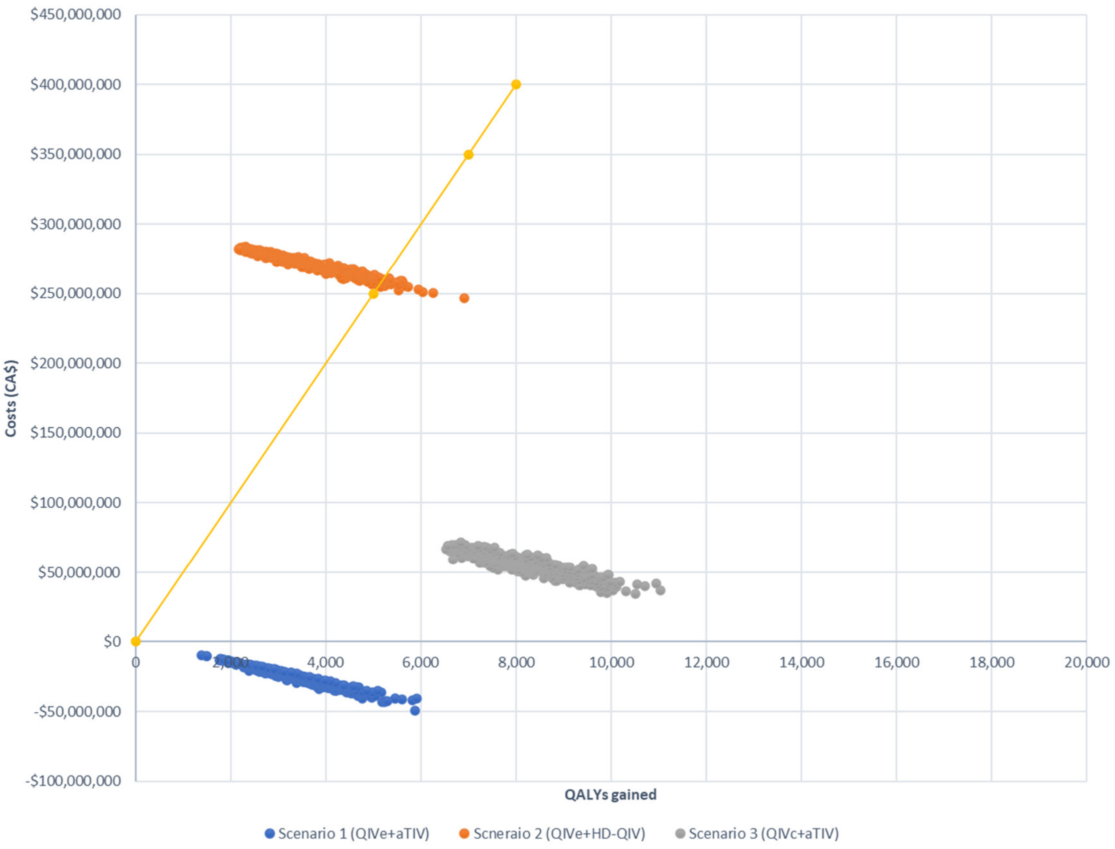

Yellow line indicates the willingness to pay threshold (CA$ 50,000)

Supplementary Figure S2. ICER acceptability curve for each of the three scenarios, assuming three egg-adapted seasons. Threshold for cost-effectiveness was CA$50,000.

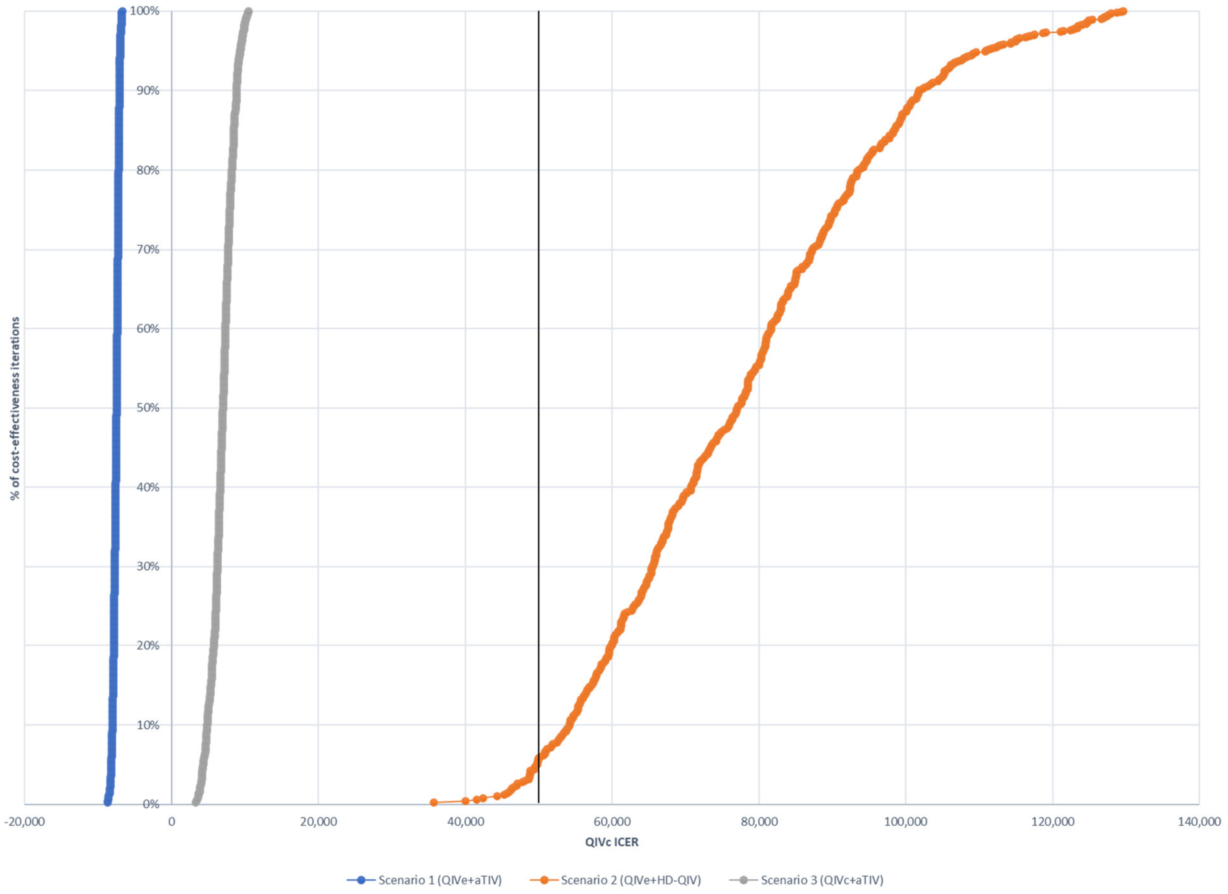

Supplement: Supplementary file 1 [file vaccines-10-01257-s001.zip › vaccines-1751189-supplementary.pdf]
